# Abnormal Venous Flow in Pregnant Women with Mild Right Ventricular Dysfunction in Repaired Tetralogy of Fallot: A Clinical Model for Organ Dysfunction in Preeclampsia

**DOI:** 10.3390/jcm14010142

**Published:** 2024-12-30

**Authors:** Anne S. Siegmund, Wilfried Gyselaers, Krystina M. Sollie-Szarynska, Tineke P. Willems, Jolien W. Roos-Hesselink, Dirk J. van Veldhuisen, Elke S. Hoendermis

**Affiliations:** 1 Department of Cardiology, University Medical Center Groningen, University of Groningen, 9700 RB Groningen, The Netherlandse.s.hoendermis@umcg.nl (E.S.H.); 2 Department of Obstetrics & Gynaecology, Ziekenhuis Oost-Limburg Genk and Faculty of Medicine and Life Sciences, Hasselt University, 3500 Hasselt, Belgium; wilfried.gyselaers@zol.be; 3 Department of Obstetrics, University Medical Center Groningen, University of Groningen, 9700 RB Groningen, The Netherlands; k.m.sollie@umcg.nl; 4 Department of Radiology, University Medical Center Groningen, University of Groningen, 9700 RB Groningen, The Netherlands; t.p.willems@umcg.nl; 5 Department of Cardiology, Erasmus Medical Center, University of Rotterdam, 3062 PA Rotterdam, The Netherlands; j.roos@erasmusmc.nl

**Keywords:** congenital heart disease, pregnancy, venous flow, right ventricular function

## Abstract

**Background:** Pregnant women with congenital heart disease carry a high risk of complications, especially when cardiac function is suboptimal. Increasing evidence suggests that impaired right ventricular (RV) function has a negative effect on placental function, possibly through venous congestion. We report a case series of hepatic and renal venous flow patterns in pregnant women with right ventricular dysfunction after repaired Tetralogy of Fallot (ToF), relative to those observed in normal pregnancy and preeclampsia. **Methods:** At 20–24 weeks pregnancy, RV function was measured by echocardiography and by cardiovascular magnetic resonance in women with repaired ToF. Combined Doppler-ECG of the hepatic and renal interlobular veins were performed in three women with asymptomatic right ventricular dysfunction. Venous impedance index and pulse transit time were measured and classified as abnormal at >75th and <25th reference percentile, respectively. **Results:** All three women showed dilated RV and mildly impaired RV function. Both hepatic and intrarenal Doppler flow waves were abnormal and very much resembled the patterns seen in preeclampsia. One of the three women had complications including ventricular tachycardia, intrauterine growth restriction, antenatal bleeding, emergency cesarean section and acute heart failure 2 days postpartum. **Conclusions:** Pregnant women with mild right ventricular dysfunction after repaired ToF show abnormal venous Doppler flow waves in the liver and kidneys, similar to those observed in preeclampsia. These findings are in line with reported observations on the association between impaired RV function, abnormal return of venous blood, venous congestion and organ dysfunction. The parallel with venous Doppler flow observations in preeclampsia suggest that the venous compartment might play an important role in the etiology of preeclampsia-induced organ dysfunction. Whether this phenomenon directly affects the uteroplacental circulation is to be assessed in future research.

## 1. Introduction

The adult population of women with congenital heart disease is growing due to major advances in both surgical and medical treatment. A significant number of them become pregnant; however, the presence of maternal heart disease is associated with an increased risk of pregnancy complications for both mother and child [1,2,3].

Increasing evidence suggests that maternal cardiac function determines placental function and thus pregnancy outcome; however, the underlying pathophysiological mechanisms remain unclear [4]. In the general population, reduced perfusion and venous congestion are the most important factors for organ dysfunction in patients with heart failure [5,6,7,8]. Here, abnormal venous Doppler flow patterns can be observed in the liver and kidneys, preceding symptoms of organ failure [9,10].

Given that the placenta serves as a temporary fetomaternal vascular organ, similar hemodynamic interactions might be present between the heart and the placenta. Our prior studies in pregnant women with various congenital heart diseases presented a consistent association between pre-existing subclinical right ventricular (RV) dysfunction and impaired uteroplacental circulation [11,12]. We hypothesized that this may result from venous congestion. To investigate this, we performed a pilot study in three asymptomatic cases of right ventricular dysfunction after repaired Tetralogy of Fallot (ToF) with documented hepatic and intrarenal venous Doppler flow assessment.

## 2. Materials and Methods

### 2.1. Study Design and Study Population

This prospective observational pilot study was conducted between 2019 and 2021. The study was carried out in accordance with the Declaration of Helsinki. The Research Ethics Committee of the participating centers approved the study protocol and written informed consent was obtained by all participating women.

Pregnant women with repaired Tetralogy of Fallot (aged ≥ 18 years), presenting before 20 weeks of gestation at the cardiology or obstetrics outpatient clinic in one of the two leading centers were asked to participate in the study. Inclusion criteria were absence of clinical signs of cardiovascular dysfunction, no medication use and availability of echocardiographic measurements before conception. Exclusion criteria were intake of cardiovascular medication, multiple pregnancies, essential hypertension, HELLP syndrome, non-hypertensive proteinuria and intrauterine growth restriction without associated maternal symptoms, or concomitant disease such as diabetes, autoimmune or liver disease.

### 2.2. RV Function Evaluation and CMR Protocol

During routine follow-up, RV and left ventricular (LV) function were evaluated by echocardiography at 20–24 weeks of pregnancy. For accurate measurement of RV function and cardiac output, cardiac magnetic resonance imaging (CMR) in left lateral position was also performed at 20–24 weeks of pregnancy [13]. According to the American College of Radiologists MR Safe Practices Guidelines, CMR (without using contrast) is considered to be safe for both the mother and fetus [14]. For the acquisition of cardiac volumes, functional parameters and flow CMR protocols and analyses have been previously published by our group [12].

### 2.3. Venous Doppler Flow Measurements

**Figure 1:**
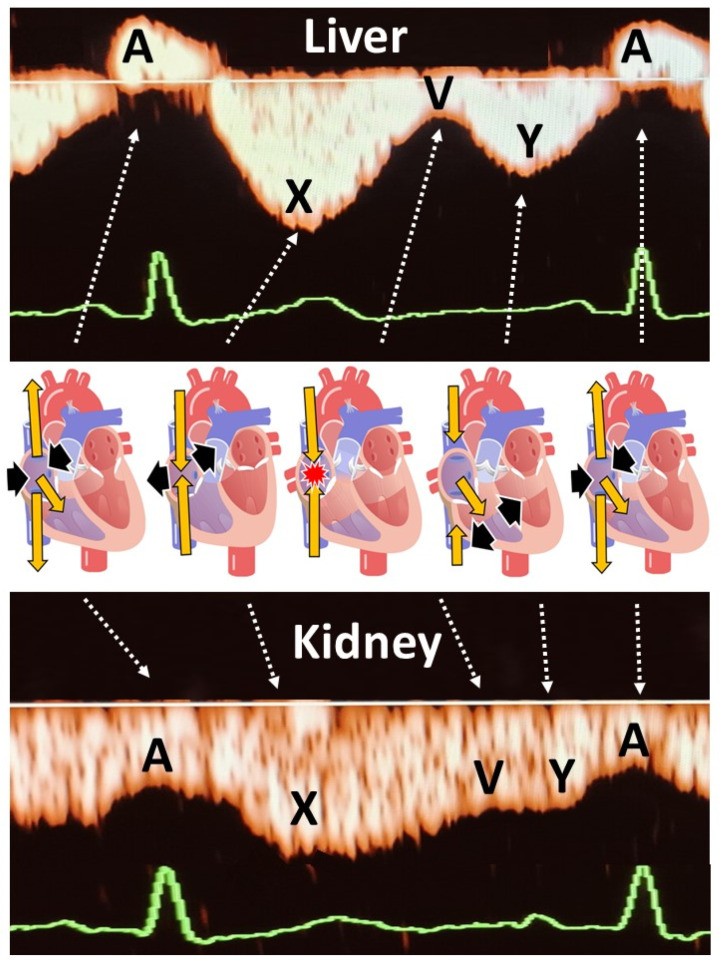
Explanation of the different venous Doppler wave deflections in the liver and kidneys, in relation to the cardiac cycle in the right atrium. A is the consequence of atrial contraction at the end of ventricular diastole. Due to the lack of valves between right atrium and vena cava, there is a retrograde jet of venous blood into the venous system causing a peak of reversed flow in the liver, and a deceleration of forward flow in the kidneys. X represents forward venous flow during right atrial diastole. V is the deceleration of forward venous flow when the right atrium is filled while the tricuspid valve is still closed. This may sometimes cause retrograde flow in hepatic veins (tetraphasic waves) as illustrated in our case series. Y represents forward venous flow during ventricular diastole with open tricuspid valve. Venous Impedance Index is defined as (X − A)/X. Venous pulse transit is defined as [time interval between ECG-P and Doppler A]/ECG R-R time interval.

At 20–24 weeks of pregnancy, non-invasive standardized Doppler flow assessment of the hepatic and renal veins were conducted as reported elsewhere [15]. Measurements included venous impedance index and venous pulse transit time (Figure 1). Venous impedance index is the Doppler equivalent of arterial resistance index, calculated as [(maximum velocity (X) minus minimum velocity (A))*/*maximum velocity (X)]. Venous pulse transit time is the heart rate corrected time interval between the P top of the ECG wave and the A wave of the Doppler pulse wave. Venous Impedance Index > 75th reference percentile and venous pulse transit time < 25th reference percentile were considered abnormal [15]. The ultrasound examination was performed by one clinician (A.S.) and analyzed offline by two clinicians (A.S. and W.G).

### 2.4. Obstetric and Neonatal Outcome

After birth, data on obstetric, maternal and neonatal outcome were obtained from the patient’s records in the referring hospitals.

## 3. Results

Results of three pregnant women with repaired ToF are shown in Table 1, relative to the pre-conceptional values (brackets). Two of them had CMR, and the third woman did not consent. All three women had hepatic and renal venous Doppler flow assessment; results are shown in Table 2 relative to the corresponding reference values as published [15].

### 3.1. Case 1

**Figure 2:**
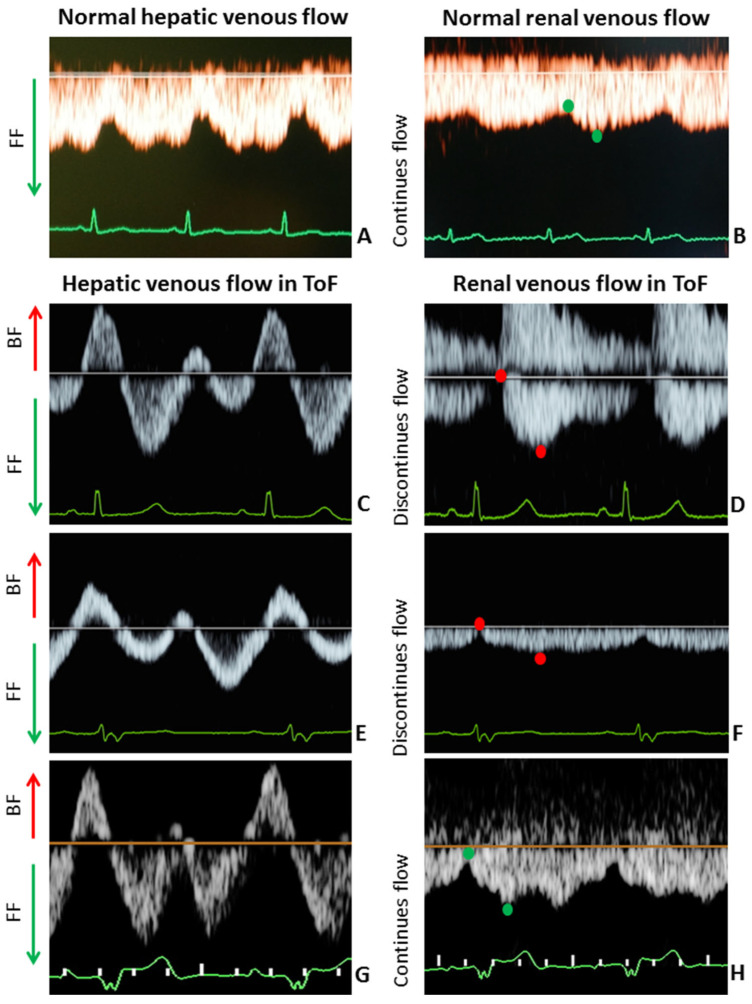
Hepatic and renal venous flow in 3 pregnant women with ToF ((**C**,**E**,**G**) and (**D**,**F**,**H**)**,** respectively) versus hepatic and renal venous flow in healthy pregnant women ((**A**) and (**B**), respectively) at 20–24 weeks gestation. Healthy controls: (**A**) + (**B**); Case 1: (**C**) + (**D**); Case 2: (**E**) + (**F**); Case 3: (**G**) + (**H**). BF, backward flow; FF, forward flow. In both liver and kidneys, backward/discontinuous flow (red arrow and red dots) were seen in pregnant women with ToF, which is in contrast to the forward/continuous flow (green arrow and green dots) in healthy controls at 20–24 weeks gestation.

A 38-year-old woman (gravida 2, para 1) underwent repair of ToF with a transannular patch at the age of 3 and a percutaneous replacement of pulmonary valve at 25 years of age. Second trimester echocardiography showed increased right ventricle (RV) dimensions and peak pressure as compared to pre-conception values. Hepatic and renal venous flow were both abnormal with elevated A-waves and pulsatile discontinuous renal flow (Figure 2C,D and Table 2). Obstetric scan parameters were normal.

Maternal ventricular tachycardia occurred at 29 + 2 weeks for which metoprolol was initiated. At 36 + 4 weeks, intrauterine growth restriction was diagnosed. At 37 + 5 weeks, emergency caesarean section was performed because of excessive blood loss. Birth weight was 2780 gr. During admission 2 days postpartum, the patient experienced acute heart failure, presenting with progressive dyspnea, pulmonary and peripheral edema and was treated with intravenous diuretics. She and her baby were discharged in good condition 4 days after delivery.

### 3.2. Case 2

A 34-year-old woman (gravida 1, para 0) underwent repair of ToF with an infundibular resection with valvulotomy at the age of 4 and a replacement of pulmonary valve at 31 years old. CMR evaluation showed RV dysfunction, mildly dilated RV and normal cardiac output. Echocardiographic measurements at midgestation were comparable to pre-conception values (6 months before pregnancy). Figure 2E,F and Table 2 show that hepatic and renal venous flow were abnormal with elevated A-wave velocities and decreased pulse transit time. Obstetric scan was normal.

Pregnancy outcome was uncomplicated, and she had a vaginal delivery at 39 + 1 weeks. The baby weighed 3355 gr, and discharge of the patient and baby took place 2 days after delivery.

### 3.3. Case 3

A 41-year-old woman (gravida 3, para 2) underwent repair of ToF with transannular patch at 0 years old and a replacement of pulmonary valve at the age of 31. CMR evaluation showed (borderline) normal RV function, dilated RV and normal cardiac output. Echocardiographic measurements at second trimester were comparable to pre-conception values (performed 2 months before pregnancy). Obstetric scan showed no abnormalities. Similar to Case 1 and 2, hepatic and renal venous flow were both abnormal with elevated atrial backflow velocities and discontinuous renal flow (Figure 2G,H and Table 2). Pregnancy outcome was uncomplicated, with vaginal delivery of a 3315 g healthy baby at 39 + 5 weeks. Both were discharged 1 day after delivery.

## 4. Discussion

The case series presented here shows for the first time that asymptomatic pregnant women with mild right ventricular dysfunction after repaired ToF show abnormal venous Doppler blood flow waves in the liver and kidneys. These waveforms are very similar to the patterns seen in women with preeclampsia. This observation supports the hypothesis that venous congestion may occur in pregnant women, even when RV dysfunction is still pre-symptomatic. This pathway offers a clue into better understanding the increased risk for preeclampsia in women with pre-conceptional right ventricular dysfunction, and—more generally—into explaining the preeclampsia-related maternal organ dysfunctions.

**Figure 3:**
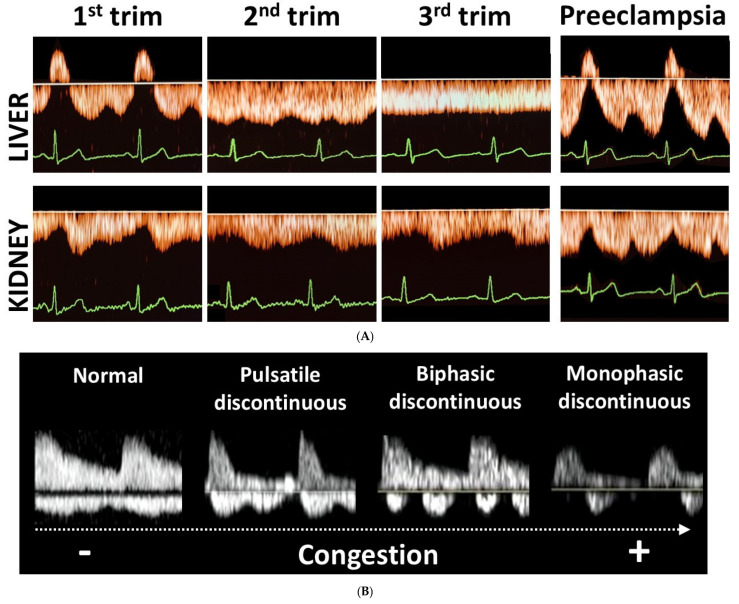
(**A**) Orange waves: Illustration of the gestational changes in hepatic and intrarenal venous Doppler waveforms from first to third trimester in normal pregnancies, and the patterns observed in preeclampsia. (**B**) White waves: Illustration of discontinuous intrarenal venous Doppler waveforms in non-pregnant individuals with increasing grade of venous congestion (Figure adapted from [19]). In pregnant women, triphasic hepatic venous Doppler waveforms become flat during the course of normal pregnancy, and biphasic intrarenal waves become monophasic. In preeclampsia, however, tri- to tetraphasic hepatic venous waveforms are present, and in the kidneys sharp decelerations of forward venous flow are observed during A- and V-waves. In the kidneys of non-pregnant individuals, discontinuous venous Doppler waves are observed, evolving from pulsatile to monophasic in parallel with degree of congestion.

As shown in Figure 2, second trimester hepatic and renal venous Doppler flow waves in all three cases are clearly different from those observed in the second trimester of uncomplicated pregnancies. Figure 3A shows the change in venous Doppler flow patterns during the course of a normal pregnancy: in the liver, a dramatic shift is observed from triphasic patterns to flat patterns, and in the kidney, biphasic waves become monophasic. This change can be explained by the physiologic increase in intraabdominal pressure during pregnancy, associated with a rise in intravascular volume and a reduction in vascular tone [15,16]. The normal gestational changes in venous Doppler flow waves also occur in women with chronic or pregnancy-induced hypertension, but not in women with preeclampsia [15]. As shown in Figure 3A, hepatic venous Doppler waves in preeclampsia are tri- or tetra-phasic, and those in the kidneys show sharp reduction in A-wave velocity as a result of retrograde rebound of right atrium contraction. One of our three pregnant women was diagnosed with intrauterine growth restriction, and none of them had preeclampsia. Despite this, they all showed (1) increased venous impedance index, (2) decreased venous pulse transit time and (3) discontinuous diastolic blood flow patterns (Table 2, Figure 2). In non-pregnant conditions, a raised venous impedance index is known to be associated with reduced RV compliance and elevated RV end-diastolic pressure [17]. In studies on patients with heart failure, this discontinuous renal venous flow is interpreted as a result of intravascular volume expansion and intermittent volume stasis with hampered drainage of venous blood from the internal organs [18,19]. The women in our case series all had increased intravascular volume as a result of physiological pregnancy-related volume expansion. Our observations demonstrate that this volume expansion, in association with asymptomatic right ventricular dysfunction, also presents with tri- to tetra-phasic hepatic Doppler waveforms and discontinuous renal venous flow. The relevance of this observation is that these abnormal venous Doppler flow patterns are considered early signs of hepatic and renal congestion [18,19]. Venous congestion is responsible for gradual deterioration in organ dysfunction and eventually organ failure, as is known to occur in acute heart failure and in cardiorenal, cardiohepatic and intraabdominal compartment syndromes [20,21,22,23]. As compared to normal pregnancy, right ventricular dysfunction and higher right ventricle systolic pressures with reduced longitudinal strain are reported in women with preeclampsia [24,25,26]. A direct link between these cardiac features and venous hemodynamics and/or organ congestion have not yet been reported. Our case series shows that right ventricular dysfunction indeed is associated with abnormal venous Doppler flow patterns, and this enhances the vulnerability to organ congestion and dysfunction [20,27,28]. Symptoms of organ dysfunction and abnormal venous Doppler waveforms distinguish preeclampsia from chronic and pregnancy-induced hypertension [15]. As such, our observation that right ventricular dysfunction is associated with abnormal venous hemodynamics and with an increased risk for preeclampsia is a clinical model that helps explain and understand the symptoms of organ dysfunction in preeclampsia.

Ever since the last decades of the previous century, the etiology of preeclampsia has been linked to a process of abnormal placentation in early pregnancy [29]. Shallow invasion of the spiral arteries by trophoblast cells is responsible for an inadequate perfusion of the intervillous space, insufficient to meet the needs of the growing conceptus. This causes a state of chronic hypoxia at the level of the placenta, with release of mediators of oxidative stress, systemic inflammation and endothelium activation in the maternal circulation, eventually causing the gestational syndrome named preeclampsia [30]. More recently, however, this theory has been expanded for several reasons. The histologic features of abnormal placentation are not pathognomonic for preeclampsia, as they are also found in the placentas of uncomplicated pregnancies and are often absent in placentas of patients with preeclampsia [31]. Clinical observations merely support abnormal uteroplacental perfusion as a consequence, rather than as a cause, of abnormal maternal circulatory function [32]. An imbalance between cardiac output and peripheral vascular resistance, already present before conception, is shown to predispose to gestational complications as preeclampsia and fetal growth restriction [33,34]. Our research group also observed a higher incidence of preeclampsia in pregnant women with congenital heart disease, specifically when right ventricular dysfunction is present [11,35]. This raises the question whether abnormal placentation may be linked to a process of backward insufficiency of the maternal circulation via a pathway of venous hemodynamic dysfunction and congestion. The case series presented here is in line with this view and is supported by the observation of peri-implantation trophoblast invasion of maternal veins as early as a month before spiral artery patency [36,37]. Whereas during the first month after embryo implantation, the lumen of the spiral arteries is blocked by trophoblast plugs, the decidual veins remain patent while they are invaded by trophoblasts [38]. This serves a direct and open communication canal between conceptus and mother already from the very first stages of implantation: extracellular trophoblast vesicles and placental microRNAs are demonstrable in the maternal serum as early as 6 weeks, whereas spiral arteries only become patent at around 8–10 weeks [39,40]. It is likely that abnormal maternal venous hemodynamics at this stage may predispose to an abnormal maternal response to trophoblast signaling, with inadequate installation of gestational adaptations of the maternal circulation that can have important clinical repercussions at more advanced stages of pregnancy. The direct link between abnormal venous hemodynamics and congestion during the process of embryo implantation and placentation is to be evaluated in future research.

### Limitations

The inclusion period was during the COVID-19 pandemic, and therefore inclusions and data collection were limited. However, since this pilot is an exploratory study, we decided to write down our first three cases, as they all showed abnormal venous flow associated with pre-existing right ventricular dysfunction. Further studies are required to assess the clinical value of hepatic and renal vein examination during pregnancy.

## 5. Conclusions

Pregnant women with right ventricular dysfunction after repaired ToF have abnormal venous flow in the liver and kidneys. These abnormalities were observed in asymptomatic patients with mildly impaired RV function. These findings are in line with the hypothesis that subclinical impaired RV function may negatively affect placental function via a pathway of venous congestion.

## Figures and Tables

**Table 1:** Echocardiography and CMR at 20–24 weeks gestation. Pre-conception echo parameters are between brackets ( ).

	Case 1	Case 2	Case 3
**Echocardiography**			
RV function			
TAPSE (mm)	22 (21)	19 (19)	18 (19)
S’ (cm/s)	15.2 (10.2)	9.1 (9.6)	-
FAC (%)	32	28	35
RV dimensions			
RVEDD (mm)	48 (43)	47 (46)	39 (42)
RV peak pressure (mmHg)	61 (59)	28 (29)	32
RA volume (mL)	67 (53)	53 (40)	63
LV systolic function			
LVEF (%)	55 (55)	52 (50–55)	55 (55)
LV diastolic function	Normal (normal)	Normal (normal)	Normal (normal)
Pulmonary valve stenosis	No (no)	No (no)	No (no)
Pulmonary valve regurgitation	Mild/moderate (no)	Mild (mild)	Mild (mild)
Tricuspid valve regurgitation	Mild (mild)	Mild	Mild (mild)
VCI diameter			
Expiration (mm)	16	20	20
Inspiration (mm)	3	4	-
% collapse	79 (55)	78 (63)	>50% (>50%)
**CMR**			
RV ejection fraction (%)	-	41	51
RV end-diastolic volume index (mL/m^2^)	-	138	149
RV end-systolic volume index (mL/m^2^)	-	82	73
LV ejection fraction (%)	-	59	59
LV cardiac output (L/min)	-	6.3	7.4
LV end-diastolic volume index (mL/m^2^)	-	97	131

FAC, fractional area change; LV, left ventricle; LVEF, left ventricular ejection fraction; RA, right atrium; RV, right ventricle; RVEDD, right ventricle end diastolic diameter; S’, systolic tissue Doppler velocity of tricuspid annular ring; TAPSE, tricuspid annular plane systolic excursion; VCI, vena cava inferior.

**Table 2:** Hepatic and renal venous flow at 20–24 weeks gestation as compared to the normal interquartile range (IQR) [15].

	Normal Values [IQR1–IQR3]	Patient 1	Patient 2	Patient 3
**Impedance Index**				
HVI	0.24–1.39	1.80 ↑	1.98 ↑	1.79 ↑
L RIVI	0.36–0.49	0.86 ↑	0.71 ↑	0.65 ↑
R RIVI	0.35–0.48	0.77 ↑	0.61 ↑	0.67 ↑
**Time**				
Liver VPTT	0.16–0.30	0.17	0.17	0.12 ↓
LK VPTT	0.27–0.36	0.29	0.19 ↓	0.18 ↓
RK VPTT	0.26–0.37	0.23 ↓	0.20 ↓	0.11 ↓

HVI, hepatic vein index; IQR, interquartile range; LK, left kidney; RIVI, renal interlobular vein index; RK, right kidney; VPTT, venous pulse transit time. For definitions of venous impedance index and pulse transit time, see Figure 1. ↑: higher than normal interquartile range, ↓: lower than normal interquartile range

## Data Availability

The datasets used and/or analyzed during the current study are available from the corresponding author on reasonable request.
